# Development of Defatted Soy Flour-Based Adhesives by Acid Hydrolysis of Carbohydrates

**DOI:** 10.3390/polym9050153

**Published:** 2017-04-25

**Authors:** Peitao Zheng, Yuqi Li, Feng Li, Yangting Ou, Qiaojia Lin, Nairong Chen

**Affiliations:** College of Materials Engineering, Fujian Agriculture and Forestry University, Fuzhou 350002, China; fafuzpt@163.com (P.Z.); yqli@chemteam.cn (Y.L.); fafulf@163.com (F.L.); fafuyt@163.com (Y.O.)

**Keywords:** adhesive, carbohydrate, hydrolysis, defatted soy flour, environmentally friendly

## Abstract

Soy-based adhesives are attracting increasing attention in recent years because they are a renewable and environmentally friendly raw material. Defatted soy flour (DSF), comprised of 50% protein and 40% carbohydrate, is the most widely used raw material for the preparation of soy-based adhesives that are unfortunately hampered by poor gluability and water resistance. In the present study, we developed a self-crosslinking approach to prepare a formaldehyde-free defatted soy flour-based adhesive (SBA). Carbohydrates in the DSF were hydrolyzed with 0% (controls), 0.5%, 1.0%, 2.0%, 3.0% and 5.0% hydrochloric acid, and cross-linked with proteins to prepare the SBA. The effect of hydrolyzed carbohydrates on the performance of the SBA was investigated, and hydrolyzed carbohydrates significantly increased the amount of reducing sugars, but decreased insoluble substances. Fourier transform infrared spectroscopy (FTIR) and X-ray photoelectron spectroscopy (XPS) analyses revealed an enhanced cross-linking structure with fewer hydrophilic groups in cured SBAs. Maillard reactions between hydrolyzed carbohydrates and proteins resulted in SBAs with better gluability, rheological properties and thermal stability than controls. Scanning electron microscopy (SEM) images showed that plywood bonded with SBA had a higher wood failure rate than controls. This approach has potential for preparing bio-adhesives with enhanced properties from other natural resources with a similar polysaccharides and protein composition.

## 1. Introduction

Wood adhesives play a dominant role in the wood fabrication industry, such as producing plywood and particleboard. Owing to traditional formaldehyde-based adhesives releasing residual harmful components [1,2], many researchers have undertaken efforts to explore bio-adhesives from renewable resources. The development of wood adhesives from natural and renewable resources such as soybean protein [3,4], tannins [5], and starch [6] has received significant attention. Soy protein, a natural polymer consisting of a wide range of functional groups (such as –NH_2_, –COOH, –OH, and –SH), has been widely used in the form of films and adhesives [7,8,9]. However, the application of soy protein adhesives is not in large wood composites such as plywood, due to its poor water resistance [10,11], low solid content, and high viscosity [12]. Heat treatment and different denaturation agents including acid, salt, alkali, urea, sodium dodecyl sulfate, hydroxymethyl phenol, neopentyl glycol diglycidyl ether, and 2-octen-1-ylsuccinic anhydride have been employed to modify proteins in order to improve the water resistance of the soy-based bio-adhesives [13,14,15,16,17,18,19]. Although such adhesives have good strength, the high price of soy protein isolates makes them a less attractive starting point for a wood adhesive. Defatted soy flour (DSF), a natural composite consisting of two main polymeric components (approximately 50% protein and 40% carbohydrate) and other minor components is much more economically competitive [20,21]. Many attempts to modify soy protein in DSF have been reported. However, modified DSF-based adhesives exhibit much lower wet strength than soy protein isolates due to the large number of carbohydrates in DSF [22].

Soy carbohydrates are the second-largest component in DSF and consist of approximately 50% non-structural carbohydrates (simple sugars and oligosaccharides), along with structural carbohydrates such as cellulose, hemicellulose, pectin and starch [23]. Steric hindrance and/or high viscosity resulting from carbohydrates can prevent the reaction between modifiers with proteins, thereby reducing the efficiency of modification [24]. Most carbohydrates in DSF are highly hydrophilic polysaccharides that decrease the water resistance of DSF-based adhesives. We recently described a DSF-based adhesive in which polysaccharides are enzymolyzed to reducing sugars, and the addition of Viscozyme L improved the water resistance [25]. However, the recalcitrance of polysaccharides to depolymerization and the high cost of enzymes are major obstacles to economically viable commercial implementation. Therefore, a more efficient and affordable route for hydrolysis is needed [26].

Acid hydrolysis of biomass for the production of sugars has been studied intensively for centuries, and is reported to be more efficient and cheaper than enzymatic hydrolysis [27]. For example, acid hydrolysis of soybean hull has been used to produce reducing sugars [28,29] that can cross-link with proteins to prepare adhesives. However, there are relatively few studies using acid to hydrolyze carbohydrates in DSF during the preparation of formaldehyde-free defatted soy flour-based adhesives (SBAs). The present work reports a self-crosslinking approach for preparing SBAs in which carbohydrates in DSF were hydrolyzed and cross-linked with proteins. Fourier transform infrared spectroscopy (FTIR), X-ray photoelectron spectroscopy (XPS), and thermogravimetric (TG) analyses were employed to characterize the mechanisms of the cross-linking reactions. The content of reducing sugars and insoluble substances in the hydrolyzed carbohydrates was measured, and the gluability and rheological properties were investigated to assess the effect of acid hydrolysis of carbohydrates on the performance of the SBA. Scanning electron microscopy (SEM) was used to directly visualize the fracture surface characteristics of plywood produced from a veneer and the resulting SBA.

## 2. Materials and Methods

### 2.1. Materials

DSF (53% *w*/*w* soy protein on a dry basis, 8.56% moisture, 0.97% crude fat, 6.20% ash) ground from defatted soy meal was a gift from Shandong Longfeng Soybean Co., Ltd. (Longkou, China). *Pinus massoniana* veneer (300 mm × 300 mm in size, 1.2–1.3 mm thickness) with a moisture content of 10–12 (wt %) was supplied by Jianyang Luban Wood Industry Co., Ltd. (Nanping, China). All chemicals and reagents used in this study were of analytical grade and obtained from Sinopharm Chemical Reagent Beijing Co., Ltd. (Beijing, China).

### 2.2. Separation of Carbohydrate and Protein Components

DSF (40 g) was stirred into distilled water (160 g) and the pH of the resulting mixture was adjusted to 8.5 with 2N NaOH. The mixture was stirred at 25 °C for 30 min, followed by centrifugation (Beckman J6HC, Fullerton, CA, USA) at 9000× *g* for 30 min at 4 °C to obtain an insoluble carbohydrate pellet. Supernatant proteins were precipitated at their isoelectric point (pH 4.5) and centrifuged at 7000× *g* for 15 min at 4 °C to obtain the soluble carbohydrate (supernatant) and protein residue (pellet) [30]. The pellet and supernatant containing insoluble and soluble carbohydrates, respectively, were transferred to a glass pressure bottle (Syhtnware, Beijing, China) and adjusted to 163.75 g of DSF carbohydrates to form a slurry for further acid hydrolysis.

### 2.3. Preparation of SBA

Hydrochloric acid (HCl) solution (25.99 g) with containing 0%, 3.65%, 7.30%, 14.60%, 21.90% or 36.5% HCl was dripped into the DSF carbohydrate slurry (Section 2.2) to hydrolyze the carbohydrates, and the resultant HCl concentration in the DSF carbohydrate slurry was 0%, 0.5%, 1.0%, 2.0%, 3.0% and 5.0%, respectively. After immersion into a magnetic stirring oil bath at 140 °C for 60 min, the hydrolyzed DSF carbohydrate slurry was cooled to room temperature, and neutralized with 2N NaOH until the pH reached 7.0. The protein residue (Section 2.2) was denatured at 35 °C using alkaline at pH 11 and added to the hydrolyzed DSF carbohydrate slurry. The pH of the mixture was maintained at 11 by adding 2N NaOH to generate the SBA slurries (SBA-0 or control, SBA-0.5, SBA-1, SBA-2, SBA-3 and SBA-5).

### 2.4. Characterization

Fourier transform infrared spectroscopy (FTIR) analysis. Uncured SBA samples were first prepared using a dry vacuum at −48 °C and at 6.5 Pa for 48 h, and cured SBA samples were overflowed from the plywood during the pressing process. Cured samples (2.0 mg) were fully milled with potassium bromide (200.0 mg) and compressed for FTIR analysis using a Nicolet 380 apparatus (Thermo Fisher Scientific, Waltham, MA, USA). Each sample was scanned 32 times from 400–4000 cm^−1^ at a resolution of 4 cm^−1^. Spectral data were processed using OPUS 5.5 software (Bruker Optik, Ettlingen, Germany) subsequently. All spectra were baseline corrected and min-max normalized.

X-ray photoelectron spectroscopy (XPS) analysis. Cured SBA powder samples were pressed at 20 MPa for 3 min for XPS analysis on an ESCALAB250 XPS apparatus (Thermo Fisher Scientific) equipped with an Al K source. For all samples, a low-resolution survey run was performed. The C1s group concentration was obtained from high-resolution spectra (each sample was scanned four times for spectrum integration) run at a pass energy of 20 eV with an increment of 0.1 eV. The binding energy scale was referenced to the charge of the C1s group at 284.6 eV. The Shirley background subtraction method was used for the fitting procedure. Curve-fitting analysis of the C1s and N1s groups was performed using the Gaussian–Lorentzian (70%/30%) curve-fitting function in XPS Peak 4.0. software.

Thermogravimetric (TG) analysis. After freeze-drying, the thermal stability of SBA samples was analyzed using a NETZSCH STA449F3 thermogravimeter (NETZSCH Co., Selb, Germany). Samples were heated from 25 to 600 °C at a heating rate of 10 °C/min under a nitrogen gas flow of 20 mL/min. 

Rheological analysis. Rheological properties of SBA samples were measured using a Haake MARS III rheometer (Haake GmbH, Karlsruhe, Germany) equipped with cone-plate geometry (2°, 35 mm diameter). All measurements were performed at 25 °C. Steady shear rheological measurements of SBA samples were conducted in a shear range of 0–400 s^−1^. 

Water-insoluble substance content. A 4.0 g sample (*m*_1_) of the received DSF carbohydrate slurry (Section 2.3) was dissolved in 40.0 g distilled water, followed by centrifugation at 9000× *g* for 10 min. Then, the precipitate was dispersed with another 40.0 g distilled water before being recentrifuged at 9000× *g* for 10 min. The solid residue was oven-dried at 103 °C until the weight (*m*_2_) remained unchanged. The water-insoluble substance content was calculated from the following equation:

Water-insoluble substance content (%) = *m*_2_/*m*_1_ × 100%

Three replicates were performed for each measurement.

Reducing sugar content. The total amount of reducing sugars was measured based on the 3,5-dinitrosalicylic acid (DNS) method, because aldehyde and ketone groups of reducing sugars reduce the yellow DNS to the orange 3-amino-5-nitrosalicylic acid [31]. Prior to testing, 10.0 g of the received DSF carbohydrate slurry (Section 2.3) was centrifuged at 9000× *g* for 10 min, and the supernatant was collected for reducing sugar analysis. The supernatant was placed in a test tube with DNS reagent (volume ratio = 1:1), and the mixture was heated in a boiling water bath for 5 min and then diluted 1:4 (*v*/*v*) with distilled water after cooling. The absorbance which is based on the standardization of glucose, was measured at 540 nm [32]. Three replicates were performed for each measurement.

Gluability. Three-layer plywood samples were made under the following conditions: 160 g/m^2^ of a SBA was spread for each veneer layer under static pressures of 1.2 MPa at 160 °C for 1.0 min·mm^−1^. After hot pressing, plywood samples were stored under ambient conditions for at least 24 h before the shear strength was tested. Shear strength was determined according to the Chinese National Standards GB/T 9846-2015. Ten plywood specimens (100 mm × 25 mm) were soaked in water at 63 °C for 3 h, then dried at room temperature for 10 min. Shear strength was measured via a MTS tensile testing machine (MTS, CMT5504, Shenzhen, China) at a crosshead speed of 10 mm/min. Microsoft Office Excel 2007 was the software used for data analysis (Tukey test).

Sol-gel test. A sol-gel test was performed according to our previous work [25,33]. The particle size of SBA samples was sieved into a 40–60 mesh before the test. After storage at 25 ± 1 °C and 70% relative humidity for 24 h, 1.50 g (*m*_1_) of SBA samples and 150.0 g distilled water were placed into a glass bottle and heated in a boiling water bath for 3 h. The contents of the bottle were washed and filtered using a sintered glass filter with an average pore size of 3 μm. The solid residue was dried in an oven at 103 °C until the weight (*m*_2_) remained constant. The sol fraction of adhesive was calculated using the following equation:

Sol fraction = (*m*_1_ − *m*_2_)/*m*_1_ × 100%

All measurements were performed three times, and average values were used for analysis.

Scanning electron microscopy (SEM) analysis. After shear strength testing, plywood specimens were oven-dried at 40 °C until a constant weight was achieved, and their fracture surfaces were then observed by SEM (Hitachi, SU8010, Tokyo, Japan) at an accelerating voltage of 1.0 kV.

## 3. Results and Discussion

### 3.1. FTIR Analysis

**Figure 1:**
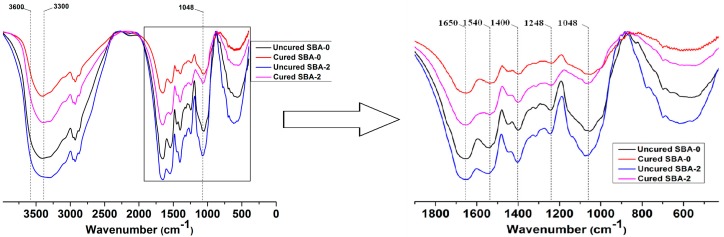
FTIR spectra of uncured SBA-0, cured SBA-0, uncured SBA-2 and cured SBA-2. FTIR = Fourier transform infrared spectroscopy; SBA = soy flour-based adhesives.

To understand the reaction mechanism between hydrolyzed carbohydrates and soy protein, FTIR spectra of uncured SBA-0, cured SBA-0, uncured SBA-2 and cured SBA-2 were compared (Figure 1). Previous studies have shown that adsorption peaks of DSF can be observed at 3300–3600 cm^−1^ (hydroxyl and amide groups, O–H and N–H stretching vibrations), 1650 cm^−1^ (amide I, C–O stretching), 1540 cm^−1^ (amide II, N–H bending), 1400 cm^−1^ and 1248 cm^−1^ (amide III, C–N stretching and N–H deformation), and 1048 cm^−1^ (hydroxyl groups bonded to carbon atoms, C–O stretching) [34]. Generally, the Maillard reaction refers to the interaction initiated between the amino groups of proteins and the aldehyde groups of reducing sugars, and this reaction may lead to a loss of NH_2_ and the formation of Amadori compounds (C–O), Schiff bases (C–N) and pyrazines (C–N) [35]. However, these products are difficult to detect in FTIR spectra because peaks can overlap in the fingerprint regions [36]. The spectrum of uncured SBA-2 included absorption peak areas for hydroxyl and amide groups, and amides I, II and III were stronger than in the spectrum of uncured SBA-0, indicating that acid hydrolysis resulted in an increase in the amount of active functional groups in the resulting SBA. Comparison with spectra from uncured SBA-2 showed that the absorption peak areas of hydroxyl and amide groups at 3300–3600 cm^−1^ and 1048 cm^−1^ in cured SBA-2 were weaker than those for uncured SBA-2, implying that active functional groups reacted with proteins in DSF. However, absorption peak areas of hydroxyl and amide groups at 3300–3600 cm^−1^ in cured SBA-2 were larger than those for cured SBA-0, which may be due to functional groups (e.g., hydroxyls) generated by hydrolysis of carbohydrates in DSF. The water resistance of the cured SBA would likely improve due to the increase in the number of cross-linkages in the structure.

### 3.2. XPS Analysis

**Figure 2:**
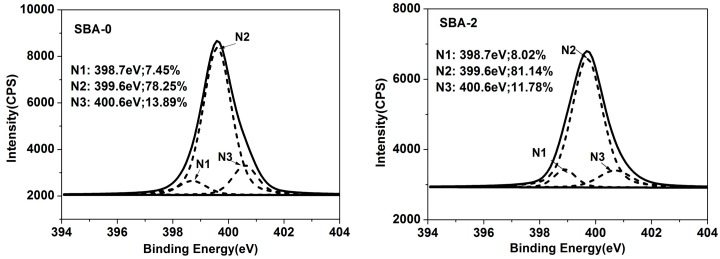

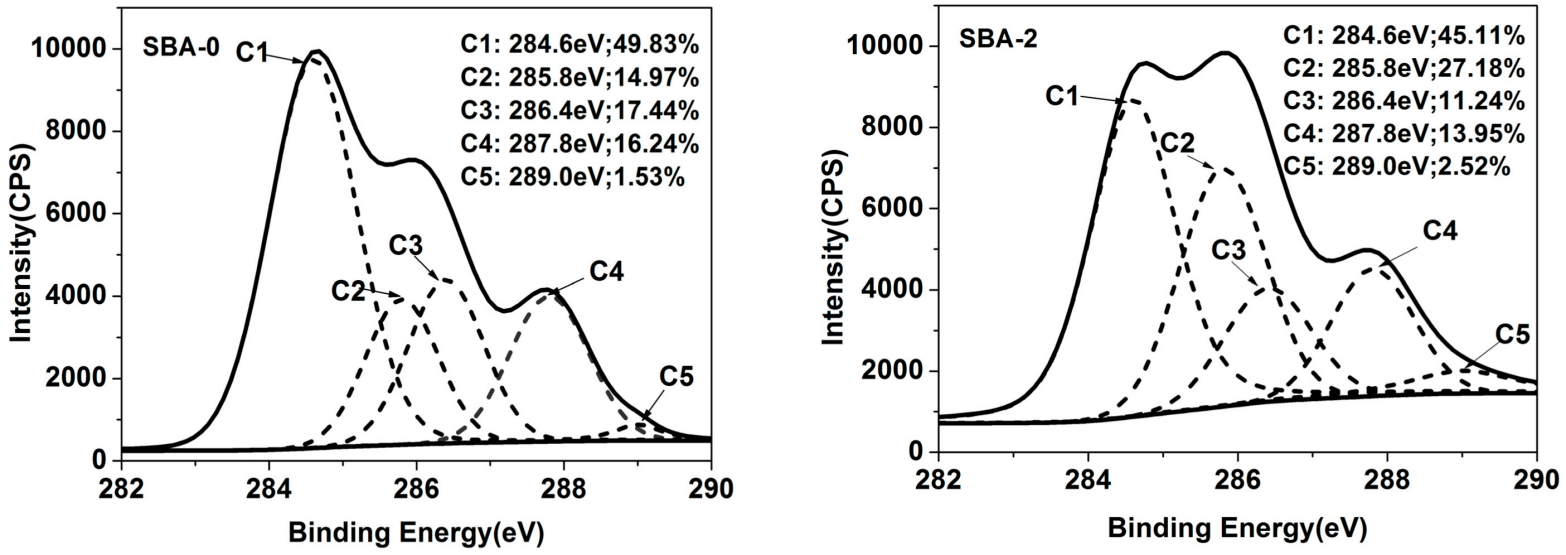
XPS spectra of cured SBA-0 and SBA-2 (N1: N–H/N–C; N2: C=N/N–C; N3: H–N–C(O)O/N–C=C; C1: C–C/C–H; C2: C–NH–C; C3: C–OH; C4: –NH–CO–/–CO(O)C–; C5:–COO–). XPS = X-ray photoelectron spectroscopy.

To further investigate the reaction mechanism, XPS analysis was performed. Nitrogen (N) in the SBA could be subdivided into three peaks; N1 (N–H/N–C), N2 (C=N/N–C), and N3 (H–N–C(O)O/N–C=C). N1 could be assigned to an amine-like N1s environment, N2 could not be resolved (reflecting the hydrophobicity of the SBA), and N3 could be assigned to the amide N category [37]. The proportion of the area under each peak, obtained from peak area computation and factor analysis, is shown in Figure 2. Compared with the cured SBA-0, an increase in the intensity of N2 in cured SBA-2 was apparent, indicating that hydrolyzed carbohydrates enhanced the Maillard reaction. An increase in the amount of N2 can contribute to the water resistance of the SBA. Carbon (C) in the SBA could be subdivided into five peaks; C1 (C–C/C–H), C2 (C–NH–C), C3 (C–OH), C4 (–NH–CO–/–CO(O)C–), and C5 (–COO–) [34]. C2 is the result of the chemical reaction of carbohydrates and proteins during the SBA curing process, reflecting the degree of cross-linking in the SBA [34]. Meanwhile, C3 and C5 reflect the hydrophilicity of the SBA. C4 arises from the presence of amide bonds in the protein component, and thus also reflects the degrees of cross-linking. It has been reported that a high water resistance for a cured adhesive is mainly the result of high hydrophobicity and cross-linking, and low hydrophilicity [38]. Compared with the cured SBA-0, the C2 content of cured SBA-2 was increased from 14.97% to 27.18%, while the content in C3 was decreased from 16.24% to 13.95%. This indicates that a greater number of Maillard reactions occurred as a result of hydrolyzed carbohydrates, in agreement with the analysis of N. Water resistance of the cured SBA can therefore be efficiently improved by increasing the degree of cross-linking and lowering the hydrophilicity. However, the decreased content of C4 and increased content of C5 in cured SBA-2 may also be related to the fact that the hydrolysis of water-soluble soy protein in DSF sugars generates functional groups such as NH_2_ and COOH.

### 3.3. Thermal Stability of SBA

**Figure 3:**
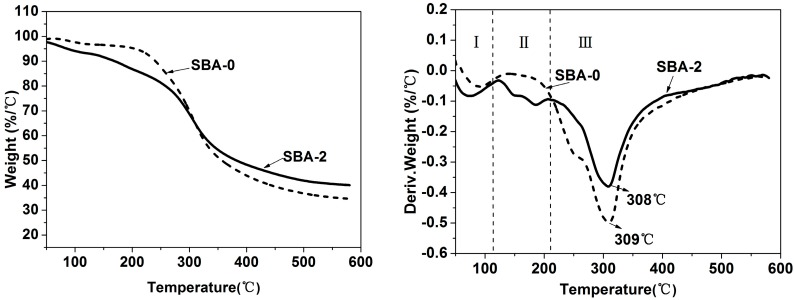
Thermogravimetric curves showing weight retention of SBA-0 and SBA-2.

Cross-linking of proteins and reducing sugars leads to a more compact network structure, which contributes to improvements in the mechanical properties of the cured SBA [36]. TGA was performed to verify the influence of cross-linking on the thermal stability of the SBA (Figure 3). As shown in Figure 3, there were three distinct stages of weight loss, which were presumably caused by the evaporation of residual moisture, and the post-curing and decomposition of adhesives, respectively [39]. After acid hydrolysis treatment, two new peaks appeared at stage II in the curve of SBA-2, which could be assigned to water generated from Maillard reactions between reducing sugars and soy protein. Although there were no obvious differences in the main decomposition temperature between SBA-0 and SBA-2, the weight loss of SBA-2 was distinctly lower. This suggests that acid hydrolysis of carbohydrates improved the thermal stability of the SBA, and further confirmed the previous FTIR and XPS analyses.

### 3.4. Rheological Properties of the SBA

**Figure 4:**
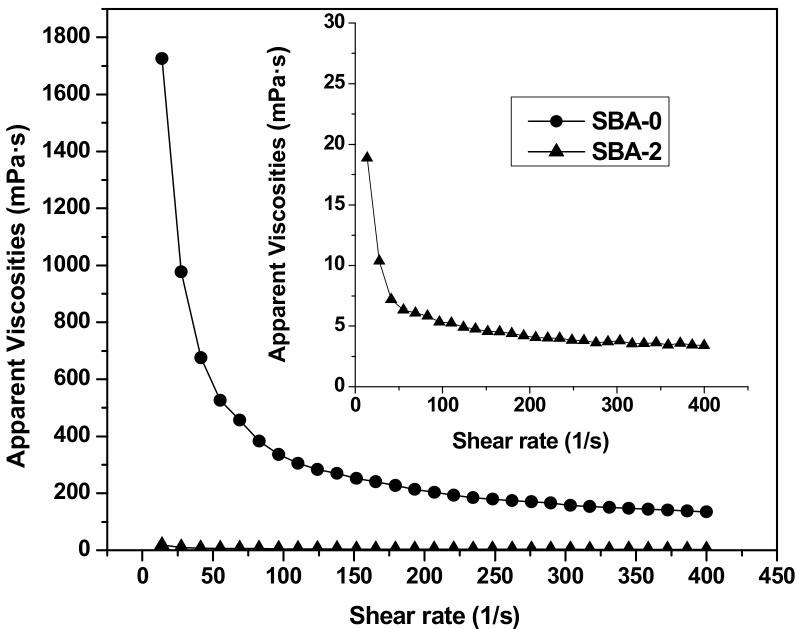
Rheological curves of SBA-0 and SBA-2.

Rheological properties play a key role in adhesive applications. Generally, adhesives with a relatively low viscosity are beneficial for handling, spreading and penetrating into the internal structure of wood [39]. The steady shear rheological properties of SBA-0 and SBA-2 are shown in Figure 4. Compared with SBA-0, the apparent viscosity of SBA-2 decreased rapidly following acid hydrolysis. Presumably, acid hydrolysis of insoluble carbohydrates in DSF increased the solubility of the resultant adhesive. The high viscosity of soy-based adhesives can restrict their potential industrial applications. For example, traditional highly alkaline soy flour adhesives have a low solids content due to viscosity limitations [40]. Besides, solid content of SBA-0 and SBA-2 were 17.43% and 17.91%, respectively. A low solids content generally requires a long press time to evaporate water, which increases the cost. Therefore, adhesives with high solids and low viscosity are needed in the wood industry. Our findings therefore offer a promising approach for decreasing the viscosity of SBAs. SBA-0 and SBA-2 both exhibited shear thinning behavior; as the shear rate increased, the apparent viscosity decreased. The relatively low viscosity at a high shear rate makes adhesives easy to mix and pour.

### 3.5. Effect of Acid Hydrolysis on Carbohydrates

During acid hydrolysis, large-chain compounds such as cellulose and hemicelluloses are broken down into oligosaccharides and depolymerized into sugars, some of which are further dissolved into water-soluble fractions [41]. Thus, insoluble substances in DSF carbohydrates were presumably decreased because water-insoluble polysaccharides were hydrolyzed to water-soluble substances, while the amount of reducing sugars increased. Reducing sugars can be dehydrated using concentrated acids [42], producing degradation by-products such as 5-hydroxymethyl-furfural and furfural [43], which in turn decreases the reducing sugar content. To determine the effect of HCl hydrolysis on carbohydrates in the DSF, the amount of reducing sugars and insoluble substances in hydrolyzed carbohydrates was measured. As shown in Table 1, the amount of insoluble substances decreased continuously with increasing HCl concentration, while the amount of reducing sugars increased dramatically between 0% and 2.0% HCl, indicating that acid treatment effectively hydrolyzed polysaccharides (insoluble substances) into reducing sugars. However, as the HCl concentration was increased from 2.0% to 5.0%, the amount of reducing sugars decreased to 2.12% following dehydration reactions. Due to the high toxicity and low chemical reactivity of degradation by-products, an HCl concentration of 2.0% may be considered an appropriate amount. These results further imply that the gluability of adhesives could be improved by Maillard reaction-mediated cross-linking between reducing sugars and proteins to form a strong three-dimensional network. 

### 3.6. Gluability and Solubility of the SBA

**Figure 5:**
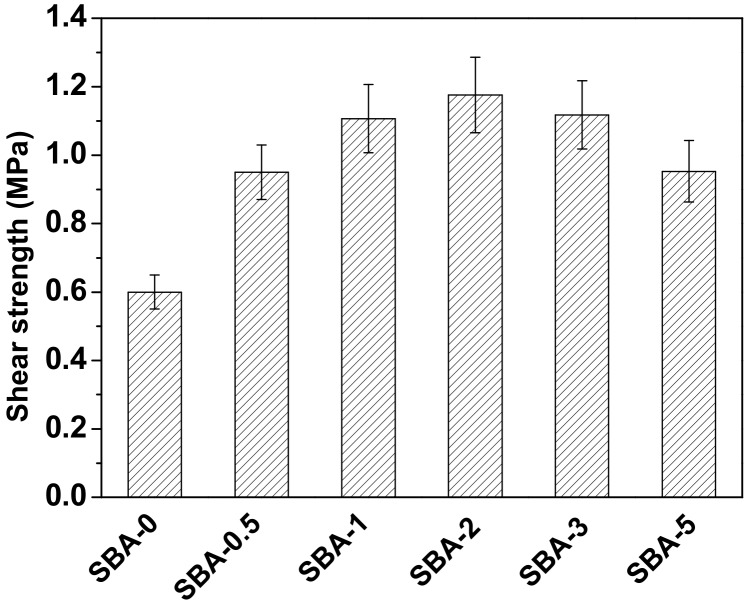
Shear strength of plywood bonded with the SBAs (Shear strength of each sample are significantly different (*p* ≤ 0.05)).

**Figure 6:**
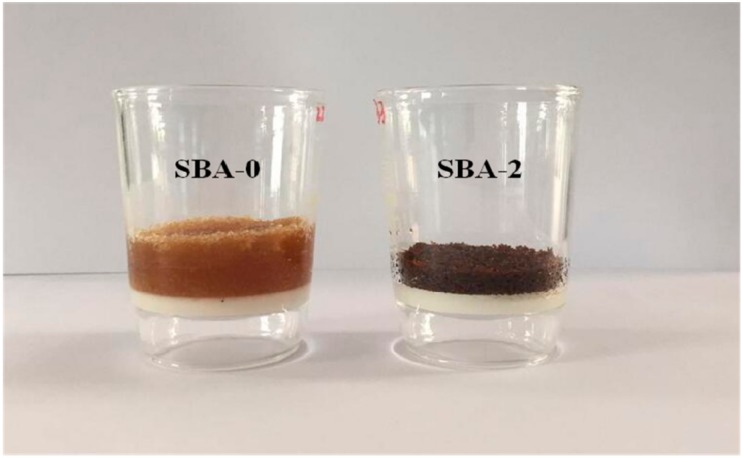
Sol–gel test of cured SBA.

Gluability of an SBA can be evaluated by measuring the shear strength of plywood bonded with SBA. The shear strength of plywood after soaking in water at 63 °C for 3 h is presented in Figure 5. Shear strength increased initially, then decreased as the HCl concentration was increased, in accordance with the observed variation in the reducing sugar content (Table 1). This could be ascribed to cross-linking between reducing sugars and proteins by Maillard reactions. It has been reported that cross-linking of proteins can improve the mechanical properties of cured adhesives [44,45]. Plywood bonded with SBA-2 possessed the highest shear strength (1.18 MPa), and achieved the highest water resistance of all SBAs in this experiment. In order to further evaluate the water resistance of the SBA, the solubility of cured SBA was studied by a sol–gel test. The filtered SBA after boiling water treatment was shown in Figure 6. Compared with cured SBA-0, cured SBA-2 showed a slight decrease in the amount of sol fraction (Table 2). However, the swelling of the cured SBA-2 was obviously lower than SBA-0, indicating that the water resistance of adhesive was improved by hydrolyzing carbohydrates in DSF. As we know, carbohydrates interact closely with proteins through hydrogen bonding, but hydrogen bonds are easily broken under wet conditions, which decreases the water resistance of SBAs. Carbohydrates from soybean can be hydrolyzed into reducing sugars that are highly reactive and readily cross-link with protein to generate a network structure that prevents water immersion and thus improves the water resistance of the resultant adhesives. The shear strength of plywood bonded with SBAs in the present study met the requirements (≥0.8 MPa) of the Chinese National Standard GB/T 9846-2015 for plywood type II, indicating suitability for interior plywood applications. 

### 3.7. Fracture Surface Analysis

**Figure 7:**
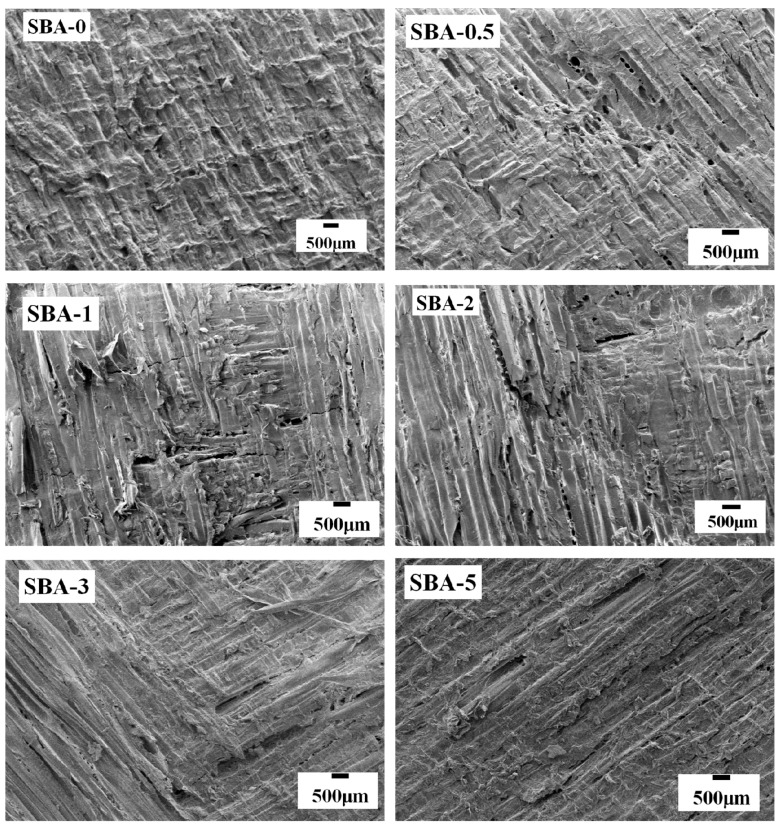
SEM micrograph of the fracture surfaces of plywood samples glued with the SBAs.

In order to assess the bonding performance of the adhesives, SEM images of the fracture surface of plywood specimens were obtained after shear strength tests. As shown in Figure 7, interfacial failure was observed in the fracture surface of plywood bonded with SBA-0, SBA-0.5, and SBA-5, suggesting that the cohesive forces of the cured SBAs were stronger than the interfacial forces between adhesives and *Pinus massoniana* wood. In contrast, SBA-1, SBA-2 and SBA-3 showed an excellent gluability to *Pinus massoniana* wood, because wood failure was observed in the plywood specimens. These results can be explained by differences in chemical reactivity, distribution and penetration of SBAs. (i) Insoluble carbohydrates are mostly polysaccharides with poor permeability in wood cells (e.g., SBA-0 and SBA-0.5); (ii) Reducing sugars are highly reactive and readily react with proteins via Maillard reactions (e.g., SBA-1, SBA-2 and SBA-3). However, some reducing sugars (e.g., glucose) act as micromolecules that readily soak into the wood, causing “drying out”, resulting in decreased gluability, as observed with SBA-5. Therefore, an optimum HCl concentration (e.g., 2.0%) for generating hydrolyzed carbohydrates can improve the gluability of an SBA. 

## 4. Conclusions

Excellent gluability and water-resistant SBAs were prepared by Maillard reaction-mediated cross-linking HCl hydrolyzed carbohydrates with soy protein in DSF. The resultant cured SBAs possessed an improved cross-linked structure with fewer hydrophilic groups and enhanced thermal stability. Meanwhile, the decreased apparent viscosity in the HCl-treated SBAs were observed. The shear strength of plywood bonded with the HCl-treated SBAs met the requirements of the Chinese National Standard GB/T 9846-2015 for plywood for interior application. HCl treatment of carbohydrates may offer an effective method for the preparation of biomass-based adhesives for interior plywood.

## Figures and Tables

**Table 1:** Reducing sugar and insoluble substance content of HCl hydrolyzed carbohydrates.

HCl Concentration (%)	Reducing Sugars (%)	Insoluble Substances (%)
0	2.13 ± 0.18	8.70 ± 0.19
0.5	5.27 ± 0.36	5.53 ± 0.15
1.0	8.94 ± 0.41	3.74 ± 0.23
2.0	12.36 ± 0.35	2.79 ± 0.14
3.0	11.89 ± 0.38	2.56 ± 0.12
5.0	10.24 ± 0.35	2.45 ± 0.07

**Table 2:** Sol fraction of cured SBA-0 and SBA-2.

Samples	Sol Fraction (%)
SBA-0	35.49 ± 0.46
SBA-2	33.52 ± 0.37
